# *Treponema pallidum* promotes macrophage polarization and activates the NLRP3 inflammasome pathway to induce interleukin-1β production

**DOI:** 10.1186/s12865-018-0265-9

**Published:** 2018-09-14

**Authors:** Li-Rong Lin, Wei Liu, Xiao-Zhen Zhu, Yu-Yan Chen, Zheng-Xiang Gao, Kun Gao, Man-Li Tong, Hui-Lin Zhang, Yao Xiao, Wen-Dong Li, Shu-Lian Li, Hui-Ling Lin, Li-Li Liu, Zan-Xi Fang, Jian-Jun Niu, Yong Lin, Tian-Ci Yang

**Affiliations:** 1 Zhongshan Hospital, Medical College of Xiamen University, Xiamen, Fujian Province China; 2 Institute of Infectious Disease, Medical College of Xiamen University, Xiamen, Fujian Province China; 3 Xiamen Fifth Hospital, Xiamen, Fujian Province China; 4 Xiamen Hospital of Traditional Chinese Medicine, Xiamen, Fujian Province China; 5 Xiamen Huli District Maternity and Child Care Hospital, Xiamen, Fujian Province China

**Keywords:** *Treponema pallidum*, Macrophage, Polarization, NLRP3, IL-1β

## Abstract

**Background:**

The involvement of inflammasome activation and macrophage polarization during the process of syphilis infection remains unknown. In this study, A series of experiments were performed using human macrophages to research the role of NLRP3 inflammasome regulation in interleukin (IL)-1β production and its influence on macrophage polarization triggered by *T. pallidum*.

**Results:**

The results showed that in M0 macrophages treated with *T. pallidum,* the M1-associated markers inducible nitric oxide synthase (iNOS), IL-1β and TNF-α were upregulated, and the M2-associated markers CD206 and IL-10 were downregulated. In addition, we observed NLRP3 inflammasome activation and IL-1β secretion in *T. pallidum*-treated macrophages, and the observed production of IL-1β occurred in a dose- and time-dependent manner. Moreover, the secretion of IL-1β by macrophages after *T. pallidum* treatment was notably reduced by anti-*NLRP3* siRNA and caspase-1 inhibitor treatment. NAC, KCl, and CA074-ME treatment also suppressed IL-1β release from *T. pallidum*-treated macrophages.

**Conclusions:**

These findings showed that *T. pallidum* induces M0 macrophages to undergo M1 macrophage polarization and elevate IL-1β secretion through NLRP3. Moreover, the process of NLRP3 inflammasome activation and IL-1β production in macrophages in response to *T. pallidum* infection involves K^+^ efflux, mitochondrial ROS production and cathepsin release. This study provides a new insight into the innate immune response to *T. pallidum* infection.

## Background

Syphilis is an infectious, sexually transmitted disease resulting from infection by *Treponema pallidum* [1, 2]. Develop lesions in compromised tissues of infected patients are a direct manifestation of the inflammatory processes triggered by *T. pallidum* [2–4]. *T.pallidum*’ pathogen-associated molecular patterns are recognized by the host immune system through pattern recognition receptors expressed by macrophages or dendritic cells, including intracellular receptors and membrane-bound [4–6]. This recognition of pathogen-associated molecular patterns activates various members of the nucleotide-binding leucine-rich receptor (NLR) family in the cytoplasm, which results in the assembly of an NLR-containing multiprotein complex that recruits and activates caspase-1 and thereby in the production of proinflammatory cytokines, including interleukin (IL)-1β, IL-18 and IL-33. In addition, IL-1β is an inflammatory marker that is commonly used to reflect activation of the NLRP3 inflammasome [7–9].

NLRP3, the most well-studied Nod-like receptor, forms a complex composed of adaptor proteins, such as apoptosis-associated speck-like protein, and the serine protease caspase-1 [4, 10]. The NLRP3 inflammasome is activated by a broad range of stimuli arising from pathogen-associated molecular patterns released during viral, bacterial, fungal, or protozoal infection [11–14]. Previous research has revealed that NLRP3 inflammasome activation induces cathepsin B release, potassium (K^+^) efflux, and reactive oxygen species (ROS) generation [15]. *T. pallidum* infection is capable of inducing IL-1β production in Kupffer cells, and the purified *T. pallidum* lipoproteins TpF1, TpN47, TmpA, and TpN15-TpN17 are associated with the induction of tumor necrosis factor α (TNF-α) [16, 17]. Our previous study have found that the proinflammatory cytokine IL-1β is mainly secreted accompanied by NLRP3 inflammasome activation in the *T.pallidum* infected rabbits [4]. However, the involvement of inflammasome activation in macrophages during the process of syphilis infection remains unknown.

In the present study, A series of experiments were performed employing macrophages derived from human monocytic cell line (THP-1) to investigate the role of NLRP3 inflammasome regulation in IL-1β production and the influence of the NLRP3 inflammasome on macrophage polarization triggered by *T. pallidum*.

## Results

### *T. pallidum* promotes macrophage polarization

**Fig. 1:**
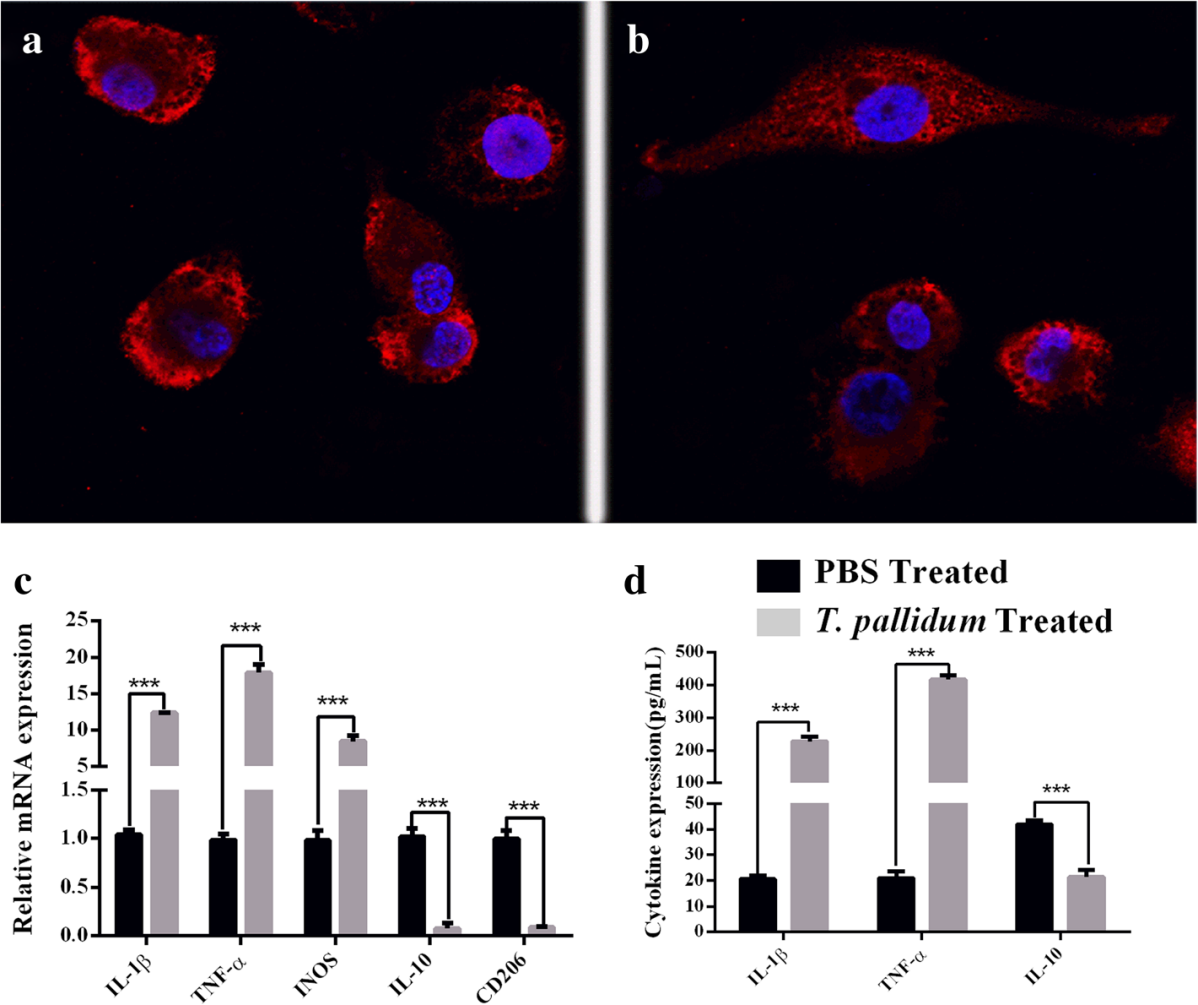
*T. pallidum* promotes macrophage polarization. **a**, THP-1 cells differentiated to M0 macrophages, and the morphological changes were observed by confocal microscopy (1000×). Nuclei were stained with DAPI (blue), and actin was stained with Alexa Fluor 633 phalloidin (red). Confocal laser scanning microscopy was conducted using an LSM700 confocal microscope (Zeiss, Oberkochen, Germany). **b**, Morphological changes in the macrophages treated with *T. pallidum* for 12 h (1000×). The image shows the typical morphological changes in the macrophages. The percentage of long orbicular macrophage cells among the macrophages after *T. pallidum* infection for 12 h was approximately 60% (data not shown). **c**, Cytokine mRNA levels in M0 macrophages treated with *T. pallidum* for 12 h. **d**, Cytokine protein levels in M0 macrophages treated with *T. pallidum* for 12 h. The results shown are from one experiment that is representative of three independent experiments and are expressed as the means±SDs. Student’s t-test was applied to compare the means between two groups. *** *P* < 0.001

THP-1 cells treated with PMA for 48 h exhibited a lack of proliferation, became attached and spread, and differentiated to M0 macrophages. The M0 macrophages were incubated with *T. pallidum* at a multiplicity of infection (MOI) of 20:1 for 12 h, and the morphology of the cells changed from suborbicular (Fig. 1a) to long fusiform with long pseudopodia, as observed by fluorescence microscopy (Fig. 1b). Macrophages treated with *T. pallidum* for 12 h showed notably increased mRNA levels of IL-1β, TNF-α and inducible nitric oxide synthase (iNOS) but decreased mRNA levels of IL-10 and CD206 compared with the levels observed in the PBS-treated control group (*p* < 0.001) (Fig. 1c). In addition, the macrophages treated with *T. pallidum* expressed significantly higher TNF-α and IL-1β protein levels but lower IL-10 levels than the PBS-treated group (*P* < 0.001) (Fig. 1d).

### *T. pallidum* promotes NLRP3 inflammasome activation and IL-1β expression in macrophages

**Fig. 2:**
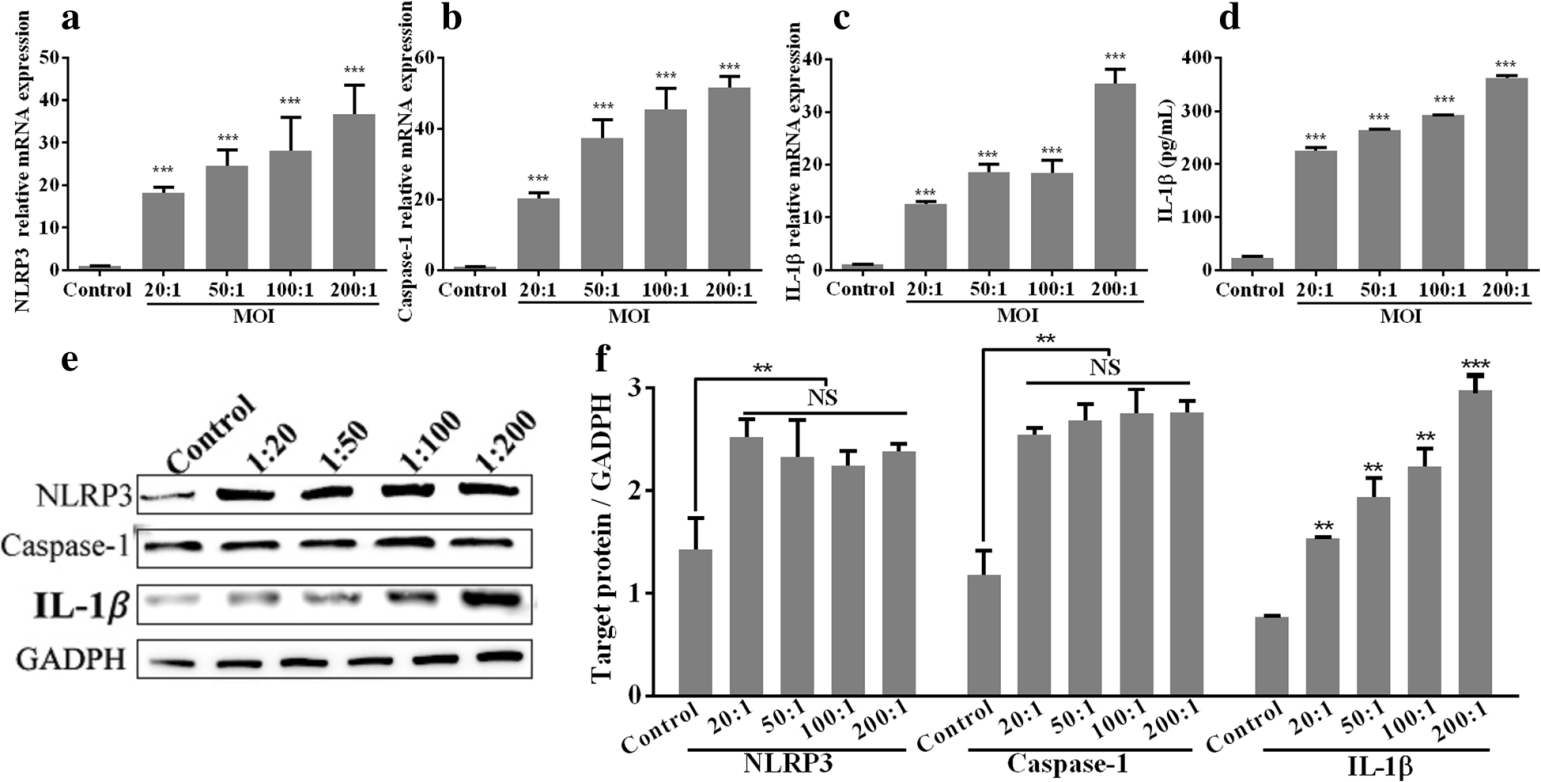
*T. pallidum* (at different MOIs) promotes NLRP3 inflammasome activation and IL-1β expression in macrophages. **a**, mRNA levels of NLRP3. **b**, mRNA levels of caspase-1. **c**, mRNA levels of IL-1β. **d**, Protein levels of IL-1β. **e** and **f**, Protein levels of NLRP3, active caspase-1, and IL-1β determined through a western blotting analysis. M0 macrophages treated with PBS served as the control group. The values represent the means±SDs of triplicate trials and are representative of three independent experiments. The western blotting results are representative of three independent experiments. Student’s t-test was used to compare the different MOI treatments with the control group. ** *P* < 0.01; ***P < 0.001

**Fig. 3:**
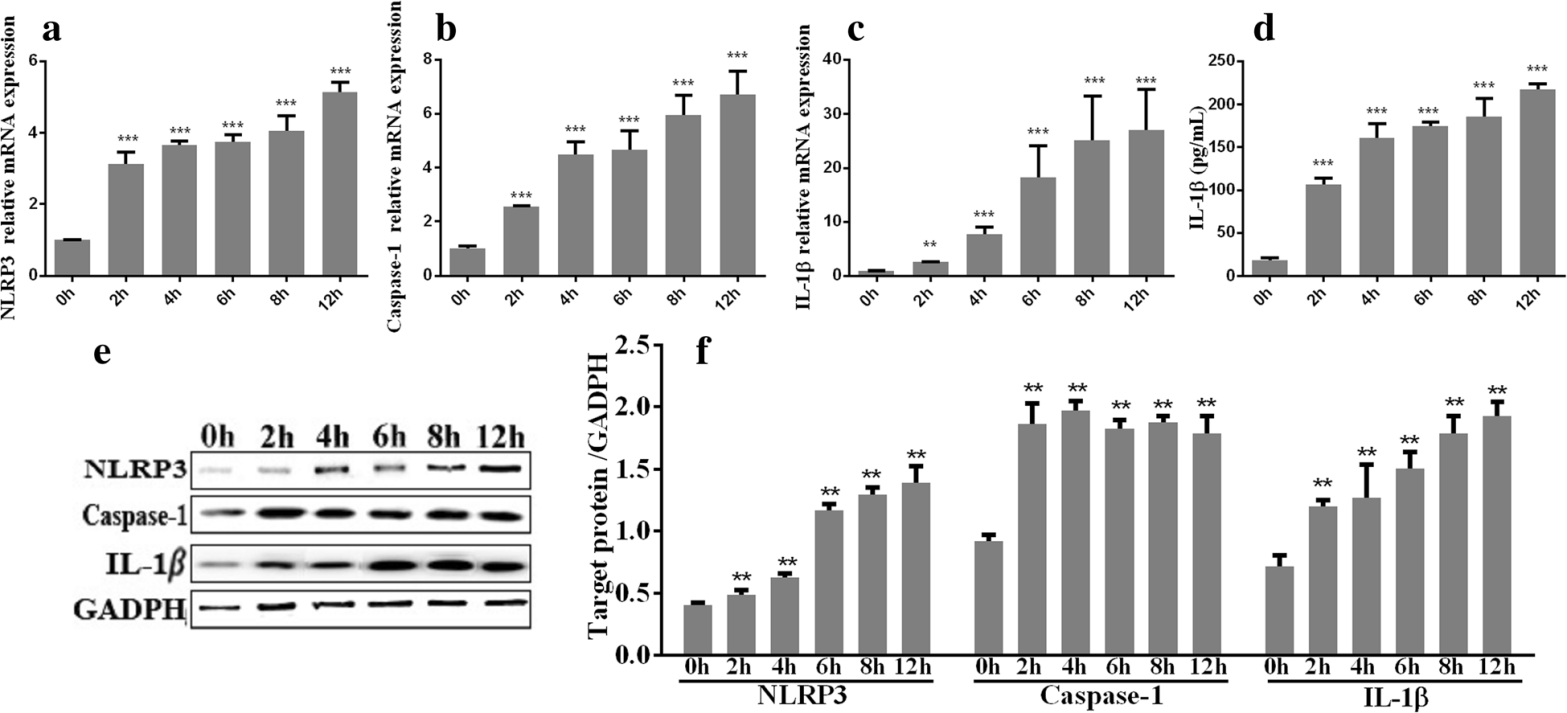
*T. pallidum* infection for different times promotes NLRP3 inflammasome activation and IL-1β expression in macrophages. **a**, mRNA levels of NLRP3. **b**, mRNA levels of caspase-1. **c**, mRNA levels of IL-1β. **d**, Protein levels of IL-1β. **e** and **f**, Protein levels of NLRP3, active caspase-1, and IL-1β determined by western blotting analysis. The values represent the means±SDs of triplicate trials and are representative of three independent experiments. The western blotting results are representative of three independent experiments. Student’s t-test was applied to compare the cells infected for different times with the cells infected for 0 h. ** *P* < 0.01; *** *P* < 0.001

The mRNA levels of NLRP3, caspase-1, and IL-1β in M0 macrophages treated with *T. pallidum* at various MOIs (20:1, 50:1, 100:1 and 200:1) for 12 h increased in a dose-dependent manner (*P* < 0.001) (Fig. 2a-c). Stimulation with *T. pallidum* also induced IL-1β protein production by the macrophages in a dose-dependent manner (Fig. 2d). In contrast, a statistical analysis showed that the NLRP3 and active caspase-1 protein levels in the treated cells were significantly higher than those in the control group, but significantly different levels were not obtained with the various MOIs (Fig. 2e, f). In addition, macrophages incubated with *T. pallidum* at an MOI of 20:1 for 0, 2, 4, 6, 8, and 12 h showed increases in the mRNA levels of NLRP3, caspase-1, and IL-1β and in the concentration of IL-1β over time (Fig. 3a-d). In addition, the concentrations of NLRP3, active-caspase-1 and IL-1β increased with increases in the incubation time (Fig. 3e, f).

### Impact of NLRP3-targeting siRNA on IL-1β expression

**Fig. 4:**
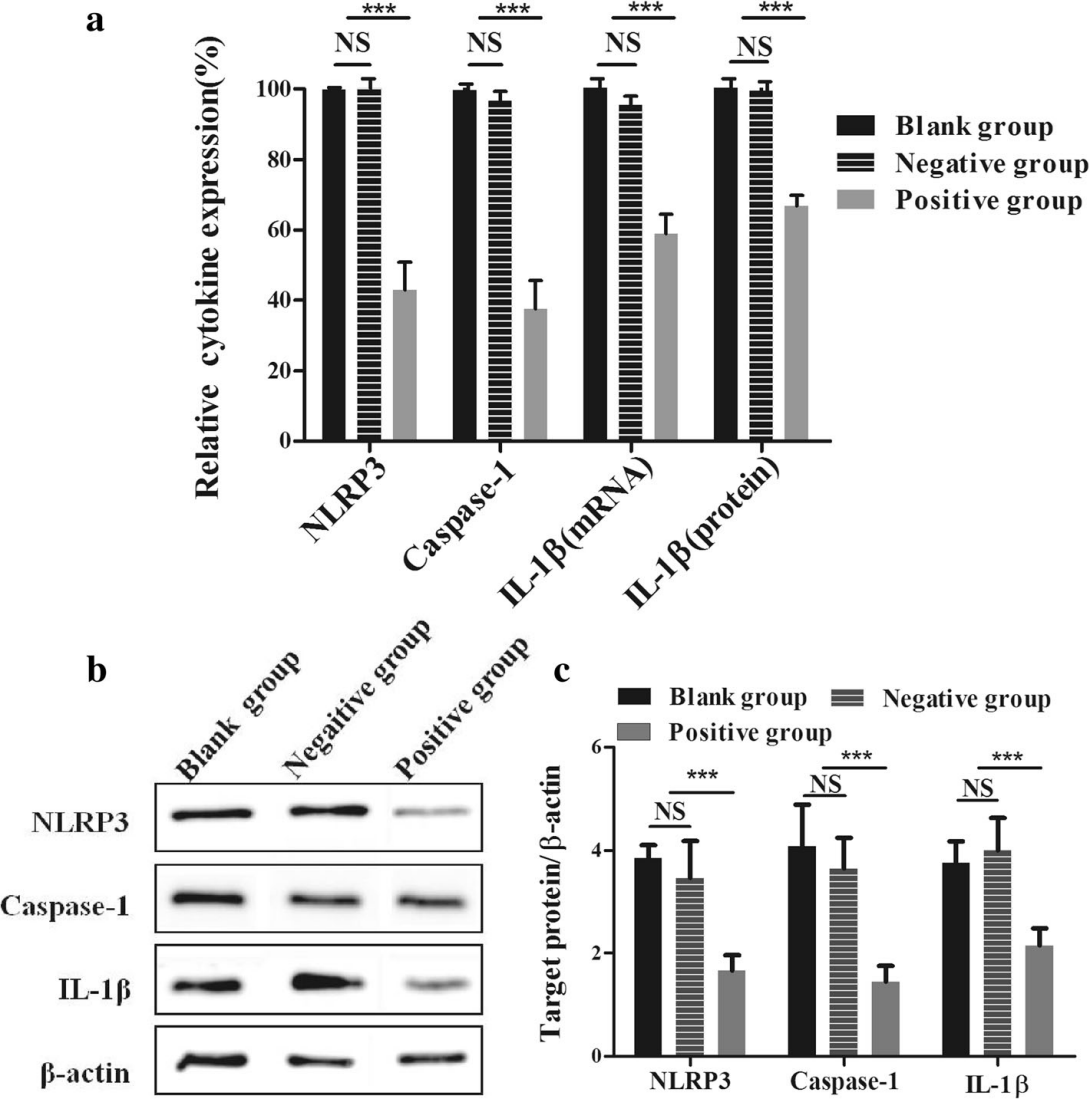
Impact of NLRP3-targeting siRNA on IL-1β expression. **a**, Inhibition rate of NLRP3, caspase-1 and IL-1β mRNA and IL-1β protein. The mRNA levels were measured by real-time PCR, and the proteins levels were measured via ELISA. **b** and **c**, Inhibitory rates of NLRP3, IL-1β and active caspase-1 proteins measured by western blotting analysis. The values represent the means±SDs of triplicate trials and are representative of three independent experiments. The western blotting results are representative of three independent experiments. One-way analysis of variance (ANOVA) was employed to examine differences between groups, and Dunnett’s post-comparison test was used to conduct multiple comparisons. NS, non-significant difference; *** *P* < 0.001

To further investigate the effect of the treatment of macrophages with *T. pallidum* on IL-1β expression and release, NLRP3 was knocked down in macrophages with anti-*NLRP3* siRNA. The results showed that 12 h after transfection, the anti-*NLRP3* siRNA group exhibited significantly (*P* < 0.001) decreased mRNA levels of NLRP3, caspase-1, and IL-1β (Fig. 4) and significantly (*P* < 0.001) reduced secretion of IL-1β. The mRNA or protein levels of NLRP3, IL-1β, and caspase-1 in the blank group were set to 100%. After siRNA transfection, the mRNA levels of NLRP3, caspase-1 and IL-1β were inhibited by approximately 57%, 62% and 41%, respectively, compared with those in the blank group, whereas the IL-1β protein levels were inhibited by 33% (Fig. 4a). Furthermore, a western blotting analysis revealed that the protein levels of NLRP3, active-caspase-1 and IL-1β were significantly reduced in the anti-*NLRP3* siRNA group (Fig. 4b, c). The above-described mRNA or protein levels were not significantly different between the blank group and the negative group.

### Inhibition of cathepsin B, ROS, K+ and caspase-1 impairs NLRP3 inflammasome activation and IL-1β expression in *T. pallidum*-infected macrophages

**Fig. 5:**
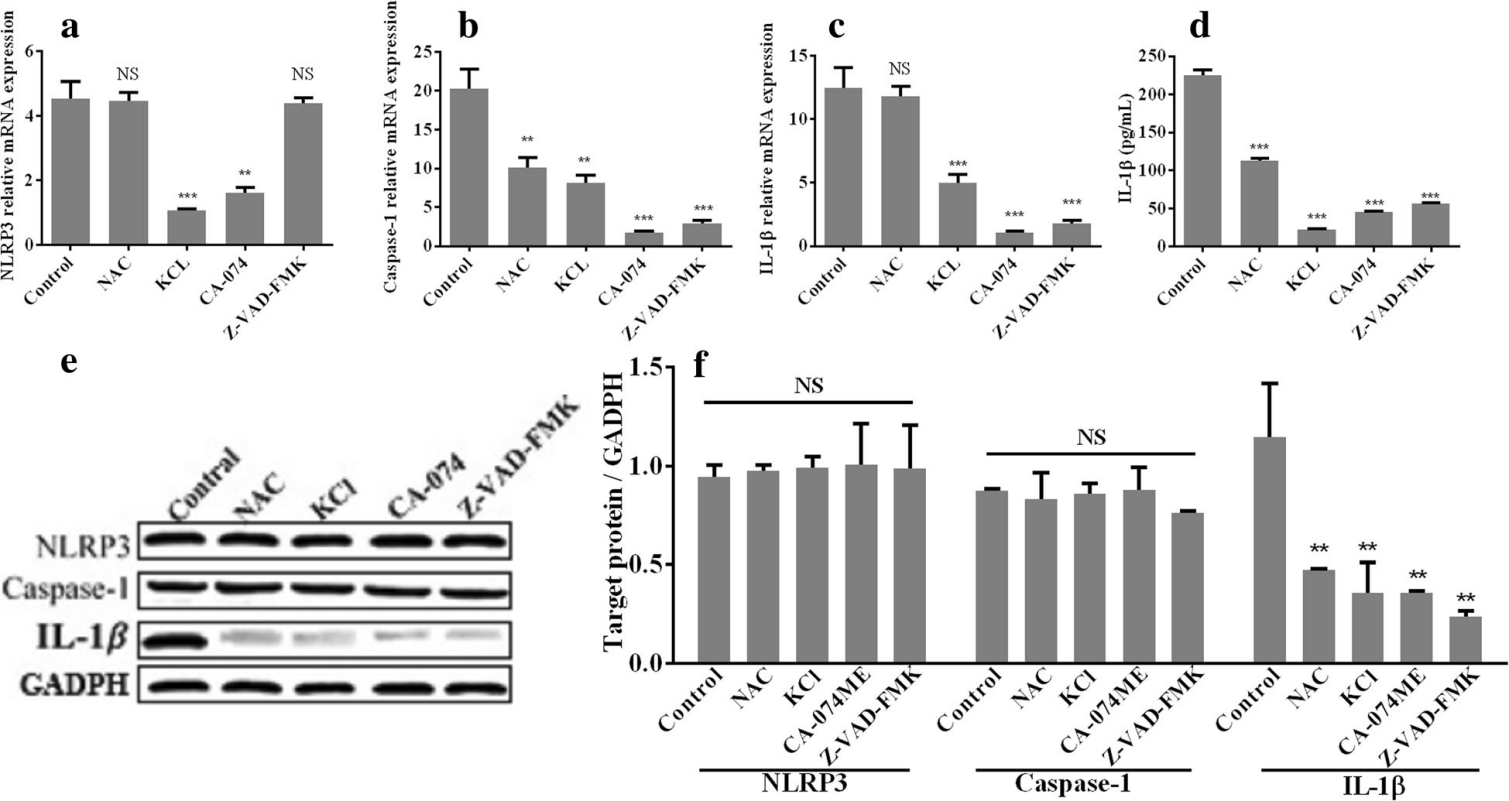
Inhibition of cathepsin B, ROS, K+ and caspase-1 impairs NLRP3 inflammasome activation and IL-1β expression in *T. pallidum*-infected macrophages. **a**, mRNA level of NLRP3. **b**, mRNA level of caspase-1. **c**, mRNA level of IL-1β. **d**, Protein level of IL-1β determined by ELISA. **e** and **f**, Protein levels of NLRP3, active caspase-1, and IL-1β determined by western blotting analysis. The values represent the means±SDs of triplicate trials and are representative of three independent experiments. The western blotting results are representative of three independent experiments. Student’s t-test was applied to compare the treatment groups with the control group. NS, non-significant difference, **P < 0.01; *** *P* < 0.001. NAC, N-acetylcysteine (an ROS inhibitor); CA074-ME, a cathepsin B inhibitor; KCl, a potassium channel inhibitor; Z-VAD-FMK, N-benzyloxycarbonyl-Val-Ala-Asp (O-methyl)-fluoromethyl ketone (a caspase-1 inhibitor)

The analysis of M0 macrophages pretreated with four inhibitors showed that the expression of NLRP3 in the macrophages treated with *T. pallidum* for 12 h was significantly inhibited by the KCl and CA074-ME pretreatments but not by the NAC and Z-VAD-FMK pretreatments (Fig. 5a), and the expression of caspase-1 mRNA was significantly inhibited by all four inhibitors, particularly CA074-ME and Z-VAD-FMK (Fig. 5b). In addition, KCl, CA074-ME and Z-VAD-FMK inhibited IL-1β at both the mRNA and protein levels, whereas NAC inhibited IL-1β at the protein level but not the mRNA level (Fig. 5c, d). Furthermore, all four inhibitors suppressed the IL-1β protein levels but had no effect on the NLRP3 and active-caspase-1 protein levels, as demonstrated in a western blotting analysis (Fig. 5e, f).

## Discussion

In our previous study, we have found that the early inflammatory response to *T. pallidum* infection is most often attributed to the local innate and adaptive cellular immune responses [3, 4]. Macrophages are the major cells in the innate immune response and play a crucial role in the early inflammatory reaction. The crucial role of macrophages in the pathogeny of syphilis was initially confirmed through a histological analysis of tissues from *T. pallidum-*infected rabbits [4, 18, 19], and subsequently, the immune sera from *T. pallidum-*infected rabbits showed markedly enhanced spirochetal clearance by peritoneal macrophages in vitro, supporting the previously described assumption [20]. The results of previous studies are generally consistent with those obtained in the rabbit model [4], and a large number of macrophages was observed in early syphilis lesions [20]. The mechanism underlying the involvement of macrophages in the early inflammatory response remains elusive. The results of our in vitro study showed that *T. pallidum* induced the polarization of M0 macrophages into M1 macrophages at the early infection phase. Within 12 h of infection, the expression of iNOS, an M1 surface marker, was upregulated, and the expression of CD206, an M2 surface marker, was decreased, which was similar with our previous study [3]. Moreover, the mRNA and protein expression levels of the M1-associated cytokines IL-1β and TNF-α and of the M2-associated cytokine IL-10 were elevated and reduced, respectively. In addition, we found that macrophages responded to *T. pallidum* infection, and this response was also accompanied by NLRP3 inflammasome activation and IL-1β secretion in vitro study.

Several types of NLRs are involved in inflammasome activation, and the NLRP3 inflammasome has been the most extensively studied of these NLRs [21]. Babolin et al. reported that TpF1 from *T. pallidum* triggers the synthesis of pro-IL-1β and inflammasome activation in monocytes, resulting in the release of IL-1β [17]. In the *T. pallidum-*infected rabbits, we found that proinflammatory cytokine IL-1β is mainly secreted accompanied by NLRP3 inflammasome activation [4]. In the present study, we confirmed that *T. pallidum* infection activated the NLRP3 inflammasome and induced IL-1β secretion in macrophages in a dose- and time-dependent manner in vitro study. The NLRP3 inflammasome, which is essential for IL-1β processing and secretion from macrophages, mediates IL-1β production in immune cells in response to *T. pallidum*. In addition, caspase-1-mediated cleavage is the limiting step in the processing of IL-1β into its secreted active forms [22]. Moreover, the knockdown of the *NLRP3* gene by anti-*NLRP3* siRNA and the inhibition of caspase-1 activation directly led to decreased expression of IL-1β in *T. pallidum-*induced macrophages. These findings suggest that *T. pallidum* activates the NLRP3 inflammasome in macrophages to induce IL-1β production in vitro study..

Several cellular events, including cathepsin release from damaged lysosomes, mitochondrial ROS generation and K^+^ efflux, have been proposed as underlying mechanisms associated with activation of the NLRP3 inflammasome [23–25]. After NLRP3 activation, caspase-1 induces IL-1β processing and secretion [5]. In the present study, M0 macrophages were pretreated with three different inhibitors and then co-cultured with *T. pallidum* to observe whether the observed effects of *T. pallidum* on caspase-1 expression and IL-1β production are inhibited by signaling inhibitors. Caspase-1 activation in the macrophages was markedly inhibited by treatment with three inhibitors, namely, KCl (a potassium channel inhibitor), NAC (an ROS inhibitor), and CA074-ME (a cathepsin B inhibitor). The production of IL-1β by the macrophages was also notably inhibited. These results confirmed that key signals, including K^+^ efflux, mitochondrial ROS generation and cathepsin release, are involved in NLRP3 inflammasome activation and IL-1β production in macrophages in response to *T. pallidum* infection.

In this study, we verified the role of NLRP3 inflammasome regulation in IL-1β production triggered by *T. pallidum* in vitro. Further studies are required to understand the roles of NLRP3 inflammasome regulation and IL-1β in *T. pallidum-*infected animals*.* In addition, although the NLRP3 inflammasome was knocked down by siRNA, all the experiments involved pharmacologic agents with inherent specificity limitations. Future studies with NLRP3- and caspase-1-knockout cell lines would provide more direct information concerning the roles of these factors in *T. pallidum* infection.

## Conclusions

In summary, our results show that *T. pallidum* induces macrophages to undergo M1 macrophage polarization and elevates IL-1β secretion through NLRP3 activation in macrophages. In addition, various signals, including ROS production, K^+^ efflux and cathepsin B release, might be involved in IL-1β production and NLRP3 inflammasome activation. Our study provides a new insight into the innate immune response to *T. pallidum* infection.

## Methods

### Preparation of macrophages

As described in our previous studies [3], THP-1 cells were obtained from the American Type Culture Collection (ATCC, Manassas, VA, USA). The cells were incubated in RPMI 1640 medium (HyClone, Logan, UT, USA) with 10% (*v*/v) heat-inactivated fetal bovine serum (Biological Industries Ltd., Kibbutz Beit Haemek, Israel), 100 U/mL penicillin and 100 g/mL streptomycin (Invitrogen/Life Technologies, Carlsbad, CA, USA). The cells were incubated in a humidified 5% CO_2_ atmosphere at 37 °C, and 5 ng/mL phorbol 12-myristate 13-acetate (PMA; Sigma-Aldrich, St. Louis, MO, USA) was then added to induce THP-1 cell differentiation into M0 macrophages for the subsequent experiments. The cytotoxicity assay was performed using an LDH kit according to the manufacturer’s instructions (Cayman Chemical Co, MI, USA).

### Effect of *T. pallidum* on macrophage polarization

The *T. pallidum* Nichols strain was kindly provided by Lorenzo Giacani, PhD (University of Washington, Seattle, WA, USA) and was propagated in rabbits as previously described [1]. Macrophages were cultured with *T. pallidum* as our previous described [3]. Briefly, macrophages were cultured with *T. pallidum* at an MOI (treponema:cell) of 20:1 for 12 h. Following treatment, cell lysates were collected for assessment of the mRNA expression of IL-1β, TNF-α, iNOS, IL-10, and CD206 by real-time PCR using the primers are listed in Table 1. The threshold cycle (Ct) of the target product was normalized to that of the internal standard glyceraldehyde 3-phosphate dehydrogenase (GADPH). The concentrations of IL-1β, TNF-a, and IL-10 in cell culture supernatants were detected using commercial ELISA kits according to the manufacturer’s recommended protocols (eBiosciences, San Diego, CA, USA). Macrophages treated with PBS were employed as the control group.

**Table 1:** Primers used for real-time PCR in this study

Genes	Primers (5′ → 3′)
NLRP3	Forward AACAGCCACCTCACTTCCAG
	Reverse CCAACCACAATCTCCGAATG
Caspase-1	Forward GCACAAGACCTCTGACAGCA
	Reverse TTGGGCAGTTCTTGGTATTC
IL-1β	Forward GATGGCTTATTACAGTGGC
	Reverse CCTTGCTGTAGTGGTGGT
GADPH	Forward GAAGGTGAAGGTCGGAGTC
	Reverse GAAGATGGTGATGGGATTTC
iNOS	Forward CAGCATCCACGCCAAGAA
	Reverse CAGGTGTTCCCCAGGTAGGTAG
TNF-a	Forward ATGAGCACTGAAAGCATGAT
	Reverse GGGCTGATTAGAGAGAGGTC
CD206	Forward GATACCTGCGACAGTAAACGA
	Reverse CTGGCTATAAGGGAATTGTGAAG
IL-10	Forward GGGTAAGCCATAAGCGAATC
	Reverse GGGCAACAAGAGCGAAACT

### NLRP3 activation and IL-1β expression in macrophages infected with *T. pallidum*

M0 macrophages (5 × 10^5^ cells/ml/well) in 2 mL of RPMI 1640 medium were cultured with *T. pallidum* at various MOIs (20:1, 50:1, 100:1 and 200:1) for 12 h, and macrophages treated with PBS were set as the control group. In addition, *T. pallidum* (1 × 10^7^ treponema/mL/well) was co-cultured with M0 macrophages (5 × 10^5^ cells/mL/well) for 0, 2, 4, 6, 8 and 12 h at 37 °C in a 5% CO_2_ incubator. Following the treatments, cell lysates were collected for the examination of mRNA and protein expression, and cell culture supernatants were used for cytokine measurements.

### NLRP3 and IL-1β mRNA expression analysis

For the assessment of mRNA expression, the total RNA from the above-described cultured cells was isolated using an RNeasy kit (Qiagen Inc., Valencia, CA, USA) and reverse transcribed with a high-capacity cDNA reverse-transcription kit (Applied Biosystems, USA). The primer pairs used for NLRP3, caspase-1, IL-1β and GADPH are listed in Table 1. The generated cDNA was amplified via quantitative PCR using TaqMan gene expression assays (Applied Biosystems, USA) with a 7500 real-time PCR system (Applied Biosystems, USA). The Ct of the target product was normalized to that of GADPH.

### NLRP3 and IL-1β protein expression analysis

The above-described cultured cells were also disrupted in 1% Nonidet P-40 lysis buffer [50 mM Tris-HCl (pH 7.4), 150 mM NaCl, 1% Nonidet P-40 (*v*/v), 2 mM EDTA, and 2 mM DTT with protease inhibitors; Roche Applied Science, USA], and the concentration of the extracted proteins was measured using a BCA protein assay kit (Takara Inc., Dalian, China). The samples were separated on 10% SDS-PAGE gels and then transferred to an Immun-Blot PVDF membrane for 1 h at 4 °C and 400 mA. After blocking with 5% milk and TBST, the membranes were incubated overnight at 4 °C with the following primary antibodies: anti-IL-1β (1:2000 dilution; Origene Inc., USA), anti-NLRP3 (1:1000 dilution; Abcam Inc., USA), anti-caspase-1 (active caspase-1(P20), 1:2000 dilution; R&D Systems Inc., USA) or anti-β-actin (1:5000 dilution; Abcam Inc., USA). The membranes were then incubated with the secondary antibody (1:5000 dilution) for 1 h, and the signals were detected using by enhanced chemiluminescence (ECL kit, Millipore Inc., USA). The IL-1β level in cell culture supernatants were also measured by ELISA as previously described.

### Impact of NLRP3-targeting siRNA on IL-1β expression

The transfection mixture comprised 3 μL of Lipofectamine 2000 (Invitrogen Inc., USA) and 50 nM anti-*NLRP3* siRNA (5’-*GGTGTTGGAATTAGACAAC-*3′; 5’-*GGATCAAACTACTCTGTGA-*3′; 5’-*GGAGAGACCTTTATGAGAA-*3′) in 500 μL of Opti-MEM (Ribo, GuangDong, China). The transfection mixture was incubated for 15 min prior to its addition to M0 macrophages. The transfected macrophages were then co-cultured with the *T. pallidum* Nichols strain (1 × 10^7^ treponema/mL/well) for 4 h, and after the addition of antibiotic-free growth medium, the culture was further incubated for 12 h. Following the treatments, cell lysates were collected for assessments of mRNA and protein expression, and cell culture supernatants were used for ELISA as described above. PBS solution (pH 8.0, 0.1 M) was used as the blank control, and cells transfected with a non-targeted control siRNA (50 nM) were used as the negative control.

### Inhibitory effects of cathepsin B, ROS, K^+^, and caspase-1 and their impact on IL-1β expression

M0 macrophages were preincubated for 30 min with various inhibitors, namely, 25 mM N-acetylcysteine (NAC) (a ROS inhibitor; Sigma-Aldrich, St. Louis, MO, USA), 30 mM CA074-Me (a cathepsin B inhibitor; Calbiochem, San Diego, CA, USA), 100 mM KCl (a potassium channel inhibitor; Sigma, St. Louis, MO, USA), or 2 μM *N*-benzyloxycarbonyl-Val-Ala-Asp(*O*-methyl)-fluoromethyl ketone (Z-VAD-FMK; a caspase-1 inhibitor; Invitrogen Inc., USA). The macrophages were then co-cultured with *T. pallidum* for 12 h as described above. Following the treatments, cell lysates were collected for the assessment of RNA and protein expression, and cell culture supernatants were used for ELISA as described above. M0 macrophages treated with *T. pallidum* but not preincubated with the inhibitors were employed as the control group.

### Statistical analysis

Student’s t-test was applied to compare the means between two groups. One-way analysis of variance (ANOVA) were used to evaluate statistically significant differences among more than two groups, and Dunnett’s post-comparison test was used to conduct multiple comparisons. A two-tailed *p* value less than 0.05 was accepted to indicate statistical significance. All the statistical analyses were performed using Prism software (version 5.0, GraphPad Software Inc., San Diego, CA, USA).

## Abbreviations

IL-1β: interleukin-1β
NLRP3: nucleotide-binding leucine-rich receptor protein 3
ROS: reactive oxygen species
